# Predicting the relationships between gut microbiota and mental disorders with knowledge graphs

**DOI:** 10.1007/s13755-020-00128-2

**Published:** 2020-11-24

**Authors:** Ting Liu, Xueli Pan, Xu Wang, K. Anton Feenstra, Jaap Heringa, Zhisheng Huang

**Affiliations:** 1 Knowledge Representation and Reasoning (KR&R) Group, Vrije Universiteit Amsterdam, Amsterdam, The Netherlands; 2 Center for Integrative Bioinformatics VU (IBIVU), Vrije Universiteit Amsterdam, Amsterdam, The Netherlands; 3 Brain Protection Innovation Center, Capital Medical University, Beijing, China

**Keywords:** Knowledge graph, Mental disorders, Neurotransmitters, Microbiota-gut–brain axis, Biomedical ontology, Gut microbiota

## Abstract

Gut microbiota produce and modulate the production of neurotransmitters which have been implicated in mental disorders. Neurotransmitters may act as ‘matchmaker’ between gut microbiota imbalance and mental disorders. Most of the relevant research effort goes into the relationship between gut microbiota and neurotransmitters and the other between neurotransmitters and mental disorders, while few studies collect and analyze the dispersed research results in systematic ways. We therefore gather the dispersed results that in the existing studies into a structured knowledge base for identifying and predicting the potential relationships between gut microbiota and mental disorders. In this study, we propose to construct a gut microbiota knowledge graph for mental disorder, which named as MiKG4MD. It is extendable by linking to future ontologies by just adding new relationships between existing information and new entities. This extendibility is emphasized for the integration with existing popular ontologies/terminologies, e.g. UMLS, MeSH, and KEGG. We demonstrate the performance of MiKG4MD with three SPARQL query test cases. Results show that the MiKG4MD knowledge graph is an effective method to predict the relationships between gut microbiota and mental disorders.

## Introduction

The microbiota-gut–brain axis used to describe the complex networks and relationships between gut microbiota and the host, which reflects the inextricable association between gut microbiota and the mental health of the host [1]. A growing body of evidence points toward that gut microbiota play a role in the development of mental disorders [2]. The composition and diversity of gut microbiota in depressed patients significantly differ from those in healthy controls [3]. Gut microbiota have been implicated in many different mental disorders, e.g. eating disorders [4] and sleeping disorders [5], in humans. The underlying theory is that gut microbiota influence the mental health of the host by regulating the level of neurotransmitters [6]. On the one hand, gut microbiota produce or modulate the production of neurotransmitters [7]. *Lactobacillus plantarum*, a lactic acid-producing bacterium, increased both serotonin and dopamine levels in germ-free mice [8]. The family of *Bacillus* and *Escherichia* generate dopamine and/or norepinephrine [9], while GABA produced by the certain species of *Lactobacillus* [6]. Gut microbiota promote the synthesis of histamine [10] and acetylcholine [11] in vivo. On the other hand, these neurotransmitters have been most studied concerning mental disorders [12]. Interruptions of serotonin and norepinephrine movement lead to depression and anxiety disorders [13]. Dopamine is another neurotransmitter linked to mental disorders, such as schizophrenia and autism spectrum disorder [14, 15]. Sleep and eating disorders are believed to be the results of interrupted passages of dopamine, norepinephrine, or GABA [16, 17]. Histamine and acetylcholine have well-established roles in the regulation of cognition disorders [18]. Additional mental disorders, such as personality disorders and sex behavior disorder, have been proven to be caused by the interrupted transfer of neurotransmitter messages [19, 20]. The imbalance of neurotransmitters is one reason for the distress or impairment of personal mental health. The associations between neurotransmitters and mental disorders, and the production of neurotransmitters by various members of the gut microbial community, suggest that gut microbiota may influence the mental health of the host by regulating the level of neurotransmitters.

We hypothesis that neurotransmitters act as ‘matchmaker’ between gut microbiota imbalance and mental disorders. Collecting and analyzing the relationships between gut microbiota and neurotransmitters, as well as neurotransmitters and mental disorders, benefit for identifying and predicting the implicit relationships between gut microbiota and mental disorders. We therefore aim to gather the disparate results in existing studies into a structured knowledge base. Knowledge graphs help to combine isolated results, giving an overview of the knowledge in an area [21]. This technique is able to connect and represent semi-structured or unstructured data in a systematic way [21, 22], and supports semantic searching, question answering, and visual decision supporting [23–25]. Currently, knowledge graphs have been widely used in medicine, such as comorbidity analysis [26], drug discovery [27], healthcare services [28], medical health status classification [29], and predicting relationships between microbes and human diseases [30].

In this study, we proposed a novel knowledge graph which we named as MiKG4MD to identify and predict the relationships between gut microbiota and mental disorders. Because of the separation of the knowledge base and the library of algorithm program, MiKG4MD is extendable by linking to future ontologies by just adding new relationships between existing information and new entities. We extended the knowledge base by integrating with existing popular biomedical ontologies, e.g. Unified Medical Language System (UMLS), Kyoto Encyclopedia of Genes and Genomes (KEGG), and Medical Subject Headings (MeSH). We demonstrate the performance of MiKG4MD with three SPARQL test cases. Results show that the MiKG4MD knowledge graph is an effective method to predict the relationship between gut microbiota and mental disorders. Constructing such a knowledge graph that gathers existing knowledge resources not only enables users to achieve semantic queries and question answering but may also be supporting medical researchers to make better decisions to implement novel therapies for various mental diseases.

## Methodology

### Data sources collection

Currently, more than 200 neurotransmitters have been identified. We here only take account of six major neurotransmitters, i.e. serotonin, dopamine, GABA, norepinephrine, histamine, and acetylcholine, which are often implicated in the pathogenesis of mental disorders. Google Scholar and PubMed literature search combined the terms ‘gut microbiota’, ‘gut flora’, ‘intestinal bacteria’, ‘neurotransmitter’, ‘serotonin’, ‘dopamine’, ‘norepinephrine’, ‘GABA’, ‘histamine’, and ‘acetylcholine’ to identify studies that investigated relationships between gut microbiota and neurotransmitters. With no limitation of study design, all relevant articles were carefully reviewed by three researchers. Finally, thirty-five articles on the regulation between gut microbiota and neurotransmitters were identified for further extraction of entities and relations. The evidence level of these studies was ranked from A to E according to their strength of the randomized controlled trial design as we presented in another paper [6]. References of the relationship between neurotransmitters and mental disorders were identified through a literature search on PubMed and Google Scholar with keywords: serotonin, dopamine, norepinephrine, GABA, histamine, acetylcholine, anxiety disorders, depressive disorder, sleep disorders, eating disorders, sex behavior disorder, personality disorder, bipolar disorder, autistic disorder, cognition disorders, and learning disorders. Due to the huge amount of references for each relationship, we limit the number of references to no more than five which we selected randomly.

**Fig. 1:**
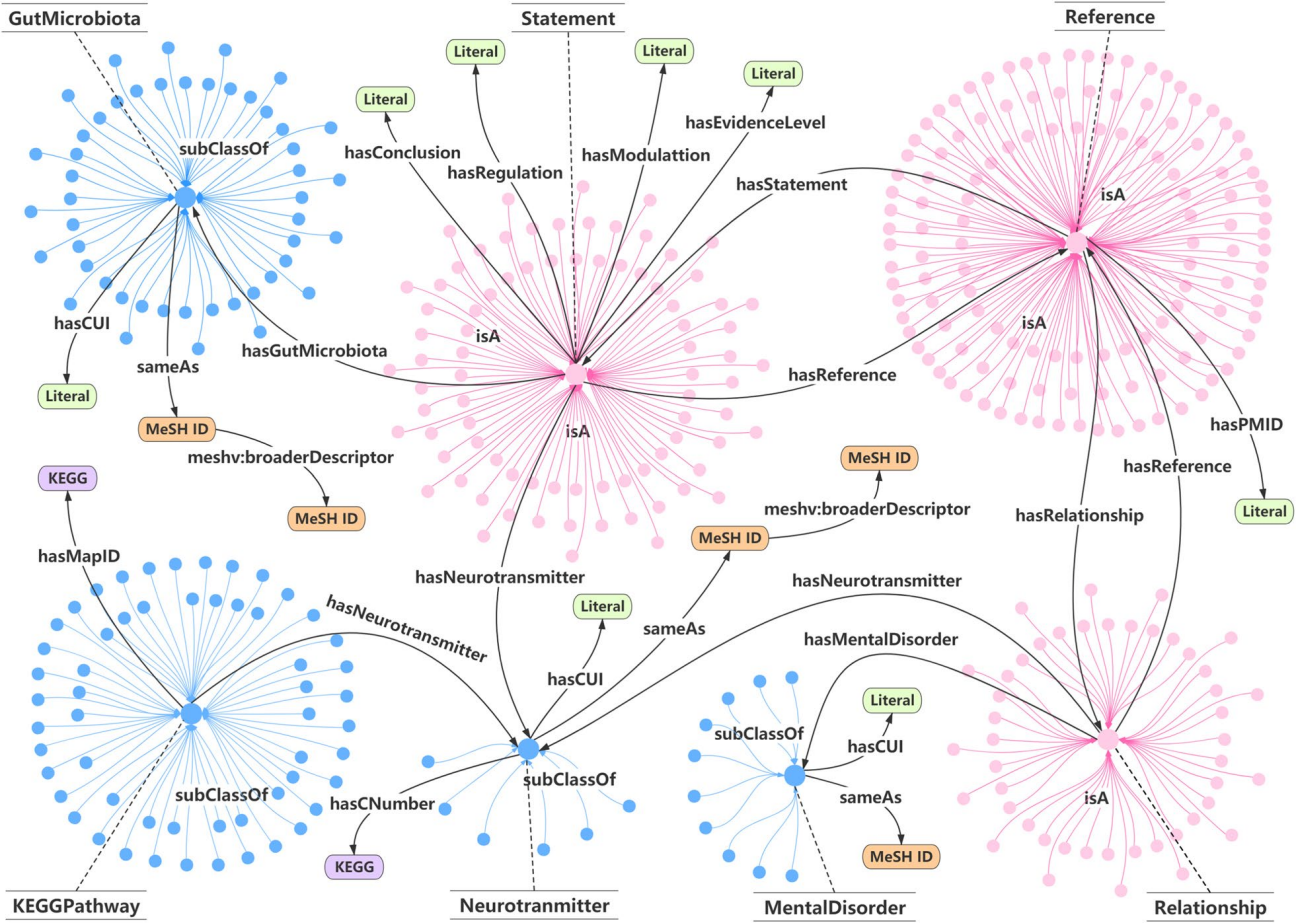
Illustration of the knowledge graph. The blue and pink circular represent TBox and ABox, respectively. Labels of edges illustrate the specific attribute and relationship. Arrows are used to show the direction of the attribute and relationship

### Data extraction and structure

To construct a knowledge graph, the most important process is to extract the entities and relations from available data sources. Many text mining tools that allow users to extract knowledge from free text, but they all require a large number of the training datasets. Even so, they often fail when encounter new terms. It was therefore decided to manually obtain highly accurate annotation of entities and semantic relations from the free text, which done by the authors. In this study, we have several classes of annotations, divided among ‘entities’ (neurotransmitter, mental disorder, gut microbiota, and KEGG pathway), and ‘relations’ (reference, statement, and relationship). The six neurotransmitters make up the class ‘Neurotransmitter’. Class ‘Mental disorders’ contains ten common mental disorders, including anxiety disorders, depressive disorder, sleep disorders, eating disorders, sex behavior disorder, personality disorder, bipolar disorder, autistic disorder, cognition disorders, and learning disorders. The forty-five entities of gut microbiota that extracted from studies constitute the class ‘Gut microbiota’. Class ‘KEGG pathway’ includes fifty-six pathways of the six neurotransmitters. Class ‘Statement’ is used to describe the semantic relational properties between gut microbiota and neurotransmitters, while ‘Relationship’ is used to describe the relations between neurotransmitters and mental disorders. We use the Terse RDF Triple Language (Turtle) format to structure the extracted entities and concepts with relations.

### Knowledge base enrichment

We enriched the semantic database by integrating with existing biomedical ontologies/terminologies, i.e. UMLS, MeSH, and KEGG databases. The UMLS is a repository of biomedical vocabularies and covers well known medical terminologies [31]. Each Metathesaurus concept in UMLS has a single Concept Unique Identifier (CUI) which links the concept data across files. The MeSH is a comprehensive controlled vocabulary thesaurus, used for indexing journal articles and books in the life sciences [32]. Each entry is identified by the MeSH Unique ID that accompanied by a definition, links to related descriptors, a list of entry terms, and role relationships in MeSH categories. In this paper, we link the concepts of gut microbiota, neurotransmitters, and mental disorders to UMLS and MeSH by matching the CUI and MeSH ID. The KEGG compound database is a collection of small molecules, biopolymers, and other chemical substances that are relevant to biological systems. Each entry is identified by the C number that contains its chemical structure and associated relationships, along with various links to other databases. KEGG pathway database is a collection of pathway maps on the molecular interaction, cellular processes, organismal systems, and human diseases [33]. Each entry is identified by the map number. Here we link the neurotransmitters with KEGG databases for further research purposes.

## Knowledge graph visualization

A knowledge graph consists of a set of concepts (classes), a set of attributes (data type properties), relationships (object properties), and constraints to abstractly represent a specific event [34]. Visualization of MiKG4MD provides a clear overview of the hierarchy and connections within the knowledge base, which is an important step in the process of knowledge graph construction. Figure 1 depicts the visualization of our knowledge graph. A knowledge base can be conceptually represented as a collection of terminologies (TBox) and assertions (ABox) [35]. TBox is used to describe a domain of interest by defining classes and properties as a domain vocabulary as shown in blue part in Fig. 1. ABox is TBox-compliant statements about individuals belonging to these sets, as shown in the pink part in Fig. 1. Nodes in rounded rectangle labeled with KEGG, Literal, and MeSH ID link internal classes with external concepts in other databases. Therefore, the constructed knowledge graph can integrate the wealth of information available from biomedical databases for semantic enrichment.

## Case study

We design three test cases to demonstrate the identification and prediction performance of the knowledge graph by using the SPARQL query. Users can perform their own queries by changing parameters as desired in templates that we designed. The MiKG4MD knowledge base, SPARQL query codes, and results of three cases are available at GitHub.^1^ Generally a SPARQL query contains three components. The PREFIX at top defines the list of ontologies that used in a query. The SELECT DISTINCT statement is used to return only distinct values. The WHERE clause is used to extract only those records that fulfill a specified condition. We use several abbreviations in the following query codes and results. ‘Ref’ is an abbreviation for reference. ‘Level’ refers to the evidence level the reference. ‘PMID’ is the unique identifier number of a reference in PubMed. Other abbreviations: GM, gut microbiota; NTM, neurotransmitter; MD, mental disorder. 
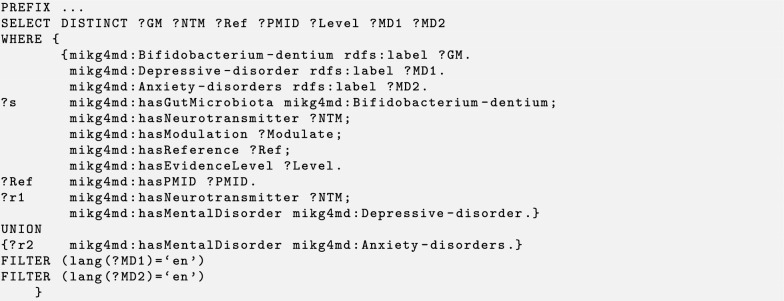

**Table 1:** The query result of test case 1

	GM	NTM	Ref	PMID	Level	MD1	MD2
1	*Bifidobacterium dentium*	GABA	DeVadder 2018	29866843	B	Anxiety disorders	Depressive disorder
2	*Bifidobacterium dentium*	GABA	Pokusaeve 2017	27458085	B	Anxiety disorders	Depressive disorder
3	*Bifidobacterium dentium*	GABA	Barrett 2012	22612585	C	Anxiety disorders	Depressive disorder

### Test case 1: gut microbiota based query

We consider a situation that a person encounters anxiety disorder and depressive disorder. We are interested in if *Bifidobacterium dentium*, an important gut bacteria of humans, may be related to anxiety disorder and depressive disorder by regulating the level of some neurotransmitters. The SPARQL query code is presented at Listing 1. In this query, *Bifidobacterium dentium* is a given condition to semantic search its related values. Therefore, we define *Bifidobacterium dentium* as the gut microbiota to query its related variables, such as neurotransmitters, reference, and mental disorder. We expect to show the reference with its PMID number, we therefore asked it after the statement. A UNION is used to combine results of anxiety disorder and depressive disorder. We apply two FILTER conditions to select labels of mental disorder in English, as shown in Listing 1. The obtained results indicate that GABA is the only neurotransmitter that modulated by *Bifidobacterium dentium*, which is supported by three references: two level B and one level C as shown in Table 1. In addition, *Bifidobacterium dentium* may relate to anxiety disorder and depressive disorder by regulating the level of GABA.
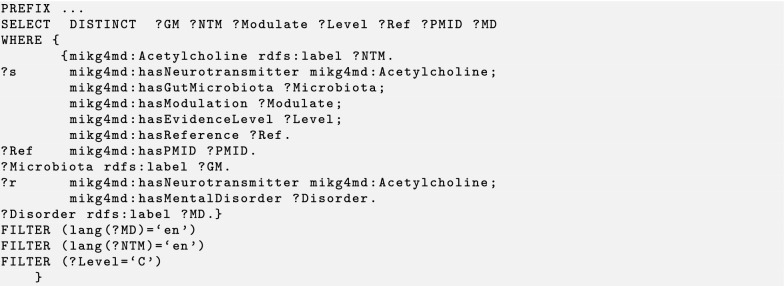

**Table 2:** The query result of test case 2

	GM	NTM	Modulate	Level	Ref	PMID	MD
1	*Lactobacillus plantarum*	Acetylcholine	Increase	C	Stanasze 1997	907345	Depressive disorder
2	*Lactobacillus plantarum*	Acetylcholine	Increase	C	Stanasze 1997	907345	Sleep disorders
3	*Lactobacillus plantarum*	Acetylcholine	Increase	C	Stanasze 1997	907345	Sex behavior disorder
4	*Lactobacillus plantarum*	Acetylcholine	Increase	C	Stanasze 1997	907345	Bipolar disorder
5	*Lactobacillus plantarum*	Acetylcholine	Increase	C	Stanasze 1997	907345	Cognition disorders
6	*Lactobacillus plantarum*	Acetylcholine	Increase	C	Stanasze 1997	907345	Autistic disorder

*Lactobacillus plantarum* associated with mental disorders by increasing the level of acetylcholine

*GM* gut microbiota, *NTM* neurotransmitter, *MD* mental disorder

### Test case 2: neurotransmitter based query

Acetylcholine serves as a primarily excitatory neurotransmitter in the central nervous system [6]. It plays a role in arousal, memory, and learning [36]. It is well known that gut microbiota generate acetylcholine. We therefore aim to investigate which gut microbiota species may be related to mental disorders by modulating acetylcholine levels. The query code we designed is shown in the Listing 2. We define the *acetylcholine* as the neurotransmitter to query its related variables, like gut microbiota, reference, PMID, and mental disorder. We show the labels of neurotransmitters and mental disorders in English by applying FILTER conditions after the WHERE clause. Besides this, we use FILTER to obtain only level C evidence in this case (Listing 2). In MiKG4MD, *Lactobacillus plantarum* is the only species of gut microbiota that modulate the acetylcholine levels, as shown in Table 2. This fact comes from one reference with level C evidence, and the PMID of the reference is 907345. The result shows that *Lactobacillus plantarum* has effects on six different mental disorders by regulating the level of acetylcholine. The six mental disorders are depressive disorder, sleep disorders, sex behavior disorder, bipolar disorder, cognition disorders, and autistic disorder.
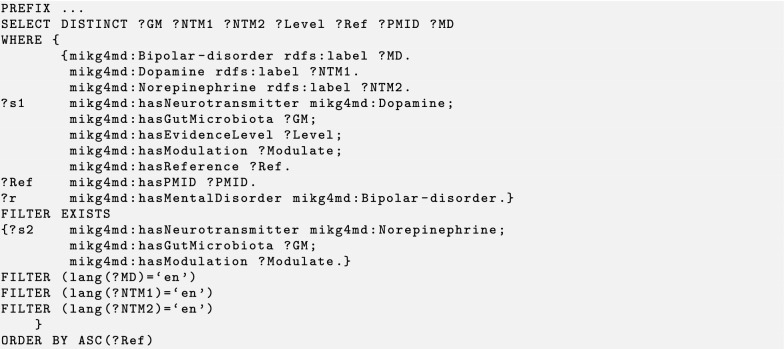

**Table 3:** The query result of test case 3

	GM	NTM1	NTM2	Level	Ref	PMID	MD
1	*Clostridium*	Dopamine	Norepinephrine	B	Asano 2012	23064760	Bipolar disorder
2	*Escherichia coli*	Dopamine	Norepinephrine	C	Shishov 2009	19845286	Bipolar disorder
3	*Bacillus mycoides*	Dopamine	Norepinephrine	C	Tsavkelova 2000	10935181	Bipolar disorder
4	*Bacillus subtilis*	Dopamine	Norepinephrine	C	Tsavkelova 2000	10935181	Bipolar disorder
5	*Escherichia coli*	Dopamine	Norepinephrine	C	Tsavkelova 2000	10935181	Bipolar disorder
6	*Proteus vulgaris*	Dopamine	Norepinephrine	C	Tsavkelova 2000	10935181	Bipolar disorder
7	*Serratia marcescens*	Dopamine	Norepinephrine	C	Tsavkelova 2000	10935181	Bipolar disorder

Some species of gut microbiota associated with bipolar disorder by affecting dopamine and norepinephrine levels

*GM* gut microbiota, *NTM* neurotransmitter, *MD* mental disorder

### Test case 3: mental disorder based query

Bipolar disorder is a serious mental disorder in which a person experiences extreme variances in mood, energy, thinking, and behavior [37]. The neurotransmitters that are implicated in bipolar disorder include dopamine, norepinephrine, serotonin, GABA, and acetylcholine [38]. We are interested in which species of gut microbiota may relate to bipolar disorder by regulating the neurotransmitter levels. For this specific condition, we here only take dopamine and norepinephrine into consideration, but not mean the others not important. We dropped the SPARQL query code at Listing 3. In this query, dopamine, norepinephrine, and bipolar disorder are the given conditions. We design the WHERE clause to find the variables according to the given conditions. At this stage, we use FILTER EXISTS to achieve the intersection result of serotonin and dopamine. FILTER conditions used to select the labels of neurotransmitters and mental disorders in English (Listing 3). As shown in Table 3, one level B evidence proved that species of *Clostridium* have effects on the content of dopamine and norepinephrine. In addition, species *Escherichia coli*, *Bacillus mycoides*, *Bacillus subtilis*, *Proteus vulgaris*, and *Serratia marcescens* modulate the levels of those two neurotransmitters. These facts come from two references with level C evidence. These species of gut microbiota strongly associated with bipolar disorder by altering dopamine and norepinephrine levels.

## Discussion and conclusion

Gut microbiota influence mental health by producing neurotransmitters directly or regulating the relevant metabolism pathways of neurotransmitters [39]. To predict the relationships between gut microbiota and mental disorders with neurotransmitters as the linking element, we first constructed a knowledge base by integrating the disparate knowledge from existing studies in a semantic way. MiKG4MD, the novel knowledge graph we proposed, enables us to identify and predict the potential connections by semantic querying and reasoning. In the process of constructing such a domain knowledge graph, relationships can be divided into explicit relationships and implicit relationships.

The support of a knowledge graph for practical applications much depends on the construction of implicit relationships. Therefore, the potential for discovering implicit relationships is crucial for a domain knowledge graph. We designed three test cases to demonstrate the potential identification and prediction performance of the knowledge graph by using the SPARQL query. The results show that gut microbiota are related to mental disorders with neurotransmitters as link. From these cases, we have learned that our knowledge graph has the potential for discovering implicit relationships. Taken together, MiKG4MD is an effective knowledge graph to identify, explore and predict the relationships between gut microbiota and mental disorders.

## Limitations and outlook

The main limitation of MiKG4MD is its limited coverage. The number of entities and relations of gut microbiota, neurotransmitters, and mental disorders are by necessity limited, which results in the query result being correct but incomplete, i.e. not covering the whole knowledge domain. As we know, gut microbiome consists of many trillions of microorganisms, including at least a thousand different species of known bacteria [40], while over 200 neurotransmitters have been identified so far [41]. Besides, there are over 300 different mental disorders listed in the DSM-5 (Diagnostic and Statistical Manual of Mental Disorders) [42]. To be sure, gut microbiota do have an impact on mental health by regulating neurotransmitters, but that is not as simple as one bacterium—one neurotransmitter. Therefore, our future work will emphasize on automated enrichment of the knowledge graph. For completely understanding the role of gut microbiota and neurotransmitters in mental disorders with a knowledge graph, like MiKG4MD, more entities and relations should be included in its knowledge base. Besides this, in the current study, we extracted entities and relations manually to ensures accuracy of the data. Manual processing, however, face huge challenges when dealing with large and wide datasets. We expect the knowledge graph would not have to be updated manually to have new information placed within it. Let the knowledge graph update itself when it collects information about entities, their properties and attributes, and relationships involving them. To realize the automation of knowledge extraction and regular updating of the database, other advanced technology (including deep learning [43–45], neuroevolution [46, 47], and evolutionary algorithms [48–50], will be employed in our future works.

## Data Availability

The data and code that support the findings of this study are openly available at GitHub (https://github.com/tingcosmos/MiKG4MD.git).

## Footnotes

1 https://github.com/tingcosmos/MiKG4MD.git
